# The Modified Stroop Task Is Susceptible to Feigning: Stroop Performance and Symptom Over-endorsement in Feigned Test Anxiety

**DOI:** 10.3389/fpsyg.2018.01195

**Published:** 2018-07-11

**Authors:** Irena Boskovic, Anita J. Biermans, Thomas Merten, Marko Jelicic, Lorraine Hope, Harald Merckelbach

**Affiliations:** ^1^Forensic Psychology Section, Faculty of Psychology and Neuroscience, Maastricht University, Maastricht, Netherlands; ^2^Department of Neurology, Vivantes Klinikum im Friedrichshain, Berlin, Germany; ^3^Department of Psychology, University of Portsmouth, Portsmouth, United Kingdom

**Keywords:** feigning, over-reporting, test anxiety, SRSI, modified Stroop task

## Abstract

Some researchers argue that the modified Stroop task (MST) can be employed to rule out feigning. According to these authors, modified Stroop interference effects are beyond conscious control and therefore indicative of genuine psychopathology. We examined this assumption using a within-subject design. In the first session, students (*N* = 22) responded honestly, while in the second session they were asked to read a vignette about test anxiety and then fake this condition. During both sessions, we administered an MST consisting of neutral, anxiety-related, and test anxiety-related words. Participants also completed the Self-Report Symptom Inventory (SRSI; Merten et al., 2016) that focuses on over-reporting of pseudosymptoms. Our feigning instructions were successful in that students succeeded in generating the typical MST effect by providing longer response latencies on anxiety related (*r* = 0.43) and test anxiety-related (*r* = 0.31) words, compared with neutral words. Furthermore, students endorsed significantly more pseudosymptoms on the SRSI (*r* = 0.62) in the feigning session than in the honest control condition. We conclude that the MST effect is not immune to feigning tendencies, while the SRSI provides promising results that require future research.

## Introduction

The modified Stroop task (MST) is widely used in research on various psychological problems. For example, the MST has been applied in investigating the cognitive underpinnings of addiction, such as alcoholism (Kramer and Goldman, 2003) or gambling (Boyer and Dickerson, 2003). Moreover, it is also used in evaluations of treatments in patients with eating disorders (Ball et al., 2004), and among sex offenders (Price and Hanson, 2007). However, the MST has been the most frequently applied in research on anxiety symptoms (see Mathews and MacLeod, 1985; Richards et al., 1992; Lovett, 2005, for reviews). In this task, participants have to name the color of neutral- and anxiety-related words as quickly as possible, while disregarding the content of the words. Typically, participants with high anxiety levels show longer reaction times for threatening than for neutral words. The MST effect is highly specific (Mathews and MacLeod, 1985) and has been documented in patients with obsessive–compulsive disorder, posttraumatic stress disorder, panic disorder, social phobia (SP), and specific phobias (Becker et al., 2001). The effect is often conceptualized as reflecting the attentional bias that anxious people have toward threatening stimuli (Mogg and Marden, 1990).

The specificity of the MST effect led researchers to test its sensitivity to intentionally feigned symptoms. For example, Buckley et al. (2003) administered the MST to actors instructed to feign PTSD, healthy controls, and patients with genuine PTSD (*N* = 18). Although the overall reaction time of actors (*n* = 6) was slower than that of healthy controls, the actors did not display the typical MST effect that was observed in the subsample of PTSD patients. It might also be important to note that in one of the previous studies, Buckley et al. (2002) did not find the specific MST effect, but rather an overall slowing down in the PTSD group (Buckley et al., 2002). However, the authors concluded that “reaction-time-based information-processing tasks such as the Stroop may be harder to fake than face valid self-report instruments” (Buckley et al., 2003, p. 64). Recent studies have drawn on this argument so as to exclude feigning as a scenario. For example, Constans et al. (2014) administered the MST to veterans with PTSD who engaged or did not engage in symptom over-reporting measured using the Miller Forensic Assessment of Symptoms Test (M-FAST; Miller, 2001). The over-reporters exhibited a stronger rather than a weaker MST effect when compared with non-over-reporters. Based on these results, the authors concluded that symptom over-reporting in their sample reflected heightened distress rather than intentional feigning. Thus, the MST effect has been used to rule out feigning.

However, Cannon (2003) pointed out that “specificity” of MST effects may reflect current concerns that people have (e.g., anxiety symptoms). For example, Mathews and MacLeod (1985) demonstrated that patients who worried mostly about physical harm were especially slow in color naming words describing physical threat, while patients who mostly worried about social threat were especially slow in color naming social threat-related words. If true, one would predict that people who feign symptoms will exhibit delayed reactions specifically for words referring to feigning (e.g., lie, fake) to the extent that they have concerns about being detected. Cannon (2003) found, indeed, that students instructed to feign mild brain trauma symptoms performed significantly worse on words pertaining to feigning relative to honest controls. With this in mind, one could attribute Buckley et al.’s failure to obtain an MST effect in actors feigning PTSD to actors’ indifference toward their task words. Perhaps, they were not familiar enough with PTSD and/or they may have found the possibility that their feigning might be detected not important.

In the current study, we wanted to test whether the MST effect occurs in students instructed to feign test anxiety. We chose test anxiety as a target for feigning because tests are an important feature of students’ lives. Thus, students have to cope with frequent examinations and due to the pressure to perform well, they often experience heightened levels of stress during tests (Lawson, 2006). Accordingly, test anxiety, i.e., a chronic preoccupation (worry) with and physiological responsiveness to test situations, is a widespread problem among students worldwide (Nelson et al., 2014). Recent studies (e.g., Yeo et al., 2016) reported that the prevalence estimates for test anxiety in students range from 10 to 40%. On the other hand, conditions such as test anxiety, dyslexia, and ADHD, which are correlated (Nelson et al., 2014), are feigned on a non-trivial scale because doing so may result in incentives (i.e., special academic privileges, such as extra time for completing exams; e.g., Musso and Gouvier, 2012). Given their experience with stressful exams, one would therefore expect that students would find it relatively easy to feign an extreme form of test anxiety.

Students who suffer from high test anxiety exhibit higher reactivity to test-related stimuli (Keogh et al., 2004). This suggests that individuals who truly suffer from test anxiety will display the MST effect. Indeed, these individuals have been found to provide longer reaction times on Stroop trials with relevant threat words (e.g., test, inept) than on trials with neutral words (e.g., MacLeod and Rutherford, 1992). In the current study, we examined whether this pattern can be simulated by non-test anxious students who feign test anxiety. To establish that participants did indeed comply with feigning instructions, we administered the Self-Report Symptom Inventory (SRSI; Merten et al., 2016). The SRSI is a recently developed measure of symptom over-reporting and includes two main scales, one consisting of genuine symptoms and the other of pseudosymptoms scale. Both scales cover a wide range of psychological (e.g., anxiety, depression) and physical (e.g., pain) complaints. People with authentic complaints endorse more genuine symptoms than pseudosymptoms, while the reverse is true for people who feign their complaints (Merten et al., 2016). We examined whether feigning instructions will cause heightened levels of pseudosymptom endorsement on the SRSI. Finally, we also included the Brief Symptom Inventory-18 (BSI-18; Derogatis, 2001) in order to screen for general distress among students.

### Hypothesis

According to the aforementioned findings, in the current study, we anticipated that the students, when not instructed about the task, would not show any abnormalities in their response latencies regardless the word type. In contrary, when instructed to feign test anxiety, we expected that students would exhibit a corresponding MST effect. Furthermore, we foresaw that the symptom endorsement on SRSI would be considerably amplified in the second session compared with the neutral, uninstructed, testing.

## Materials and Methods

### Participants

Using the effect size (*Cohen’s d* = 1.15^1^) reported by Buckley et al. (2003) and with α set at 0.05 and β at 0.95, the lower bound sample size was found to be 13. Originally, the study included 28 participants from Maastricht University and Hogeschool Zuyd, Netherlands. However, to ensure that participants did not in any way suffer from genuine test anxiety or any other closely related form of anxiety (e.g., social anxiety; Muris, 2002), potential participants were prescreened. People with high test anxiety typically report high trait anxiety levels, which reflects relatively stable general anxiety proneness (Spielberger, 1972; Keogh and French, 2001). Thus, we administered the Spielberger State-Trait-Anxiety-Inventory (STAI-T; Spielberger et al., 1970), using a cut-off score of 46 (see Fisher and Durham, 1999). Potential participants who scored above that cut-point were excluded. The mean STAI score was 33.85 (*SD* = 6.95), and two participants were excluded. We decided to include all the participants who successfully passed the pre-screening for two reasons: First, the robustness of statistical tests usually requires a minimum of 20 participants per cell, and second, we could not predict how many participants would proceed with the study, and how many would withdraw their participation. As an additional check on high distress symptoms among participants, we administered the BSI-18 (see below). Four participants scored above the cut-off point of 11, and they were also excluded from further analysis.

The final sample included 22 undergraduates (14 women). Their mean age was 21 years (*SD* = 1.73). Students were given two vouchers of €7.5 euros each, or two credit points. The study involved two sessions, each session lasting about 1 h. The study was approved by the standing ethical committee of the Faculty of Psychology and Neuroscience, Maastricht University (ECP-159 03 12 2015).

### Measurements

#### Brief Symptom Inventory-18 (BSI-18; Derogatis, 2001)

The Dutch version of the BSI-18 is designed to measure general distress and is often used as a screen for psychological problems (Meijer et al., 2011). Typical items are: “I feel like I am going to faint,” “I feel worthless,” and “I am so restless that I can’t sit still.” Respondents evaluate items on a 0 (not at all) to 4 (very much) Likert scale. Total scores range from 0 to 72, with higher scores indicating higher general distress (Meijer et al., 2011). Cronbach’s alpha of the BSI-18 in the current study was 0.72. We employed a cut-off of 11 in order to eliminate participants with clinically significant levels of distress (De Beurs, 2011).

#### The Self-Report Symptom Inventory (SRSI; Merten et al., 2016)

The SRSI consists of two main scales with 50 symptom items each. One scale lists genuine symptoms (e.g., “I am often exhausted”), while the other scale lists pseudosymptoms (e.g., “On some days my left arm is good for nothing, on other days the right one is useless”). Both main scales include five subscales that gauge plausible (potentially genuine) or unlikely (probably non-authentic) manifestations of cognitive complaints, depression, pain, somatic, and anxiety/posttraumatic stress disorder symptoms. The rationale behind the SRSI is that honest participants/patients will endorse more genuine symptoms than pseudosymptoms, whereas this differential endorsement pattern will be absent in people who feign complaints. Previous studies on the psychometric merits of the SRSI (e.g., Merten et al., 2016) found supportive evidence for this rationale. For both scales total scores range from 0 to 50. For the pseudosymptoms scale, a cut point of 9 has been proposed (Merten et al., 2016). At this cut-point, sensitivity is 0.80 and specificity is 1.00. In the current study, Cronbach’s alpha for the full scale was 0.76 (first session; T1) and 0.93 (second session; T2). For the bogus symptoms, a cut point has been proposed of 9 (Merten et al., 2016). At this cut point, sensitivity is 0.80 and specificity is 1.00, which is probably an overestimation caused by using a non-clinical sample (van Impelen et al., 2014).

#### The Modified Stroop Task

This task was created using an E-prime application, version 2.0.10.353 (see pstnet.com), and its word stimuli were presented on a computer screen (41.1 by 40.2 cm) to the participants. Words were presented 1000 ms after a fixation cross in the center of the screen, and participants had unlimited time to provide a response. The response was given by clicking on a particular letter on the keyboard that corresponded with one of three word colors (blue, green, and red) on the screen. The reaction time was measured in milliseconds (ms; measurement error = 1 ms). We included three groups of words: neutral, anxiety-related, and test anxiety-related (see Supplementary Table 1). Anxiety-related words were derived from Becker et al. (2001), test anxiety-related words taken from Lawson (2006), whereas neutral words were derived from both articles (for more details, see the *Supplementary Material*). Each word was presented three times, in a different order, and a different color. Participants were instructed to react as fast as possible to the colors of the word and to ignore its content. The total number of trials was 108 (12 words × 3 word groups × 3 colors). Prior to the experiment trials, participants were presented with 15 (5 words × 3 colors) practice trials with neutral words (e.g., Belt, Candle, Map). The reaction time for neutral, anxiety-related, and test anxiety-related words per condition was calculated as the average response latency to all stimuli presented from the corresponding category of words.

### Procedure

We used a within-subject design in order to investigate whether a non-symptomatic sample is able to produce the MST effect considered to reflect genuine test anxiety complaints. To avoid carry-over effects, we always started with the honest session and approximately 1 week later the feign session took place. During the first session (T1), participants were instructed to respond honestly to the BSI-18 and the SRSI. They were also presented with the MST containing neutral, anxiety-related, and test-related words, and asked to name the color of the word as fast as possible, without focusing on the meaning of the words. Participants were first given the opportunity to practice with the task. After 15 practice trials, participants completed 108 active trials. During the second session (T2), participants were first given a vignette that described the following scenario (see the Supplementary Material for English version^2^): a student with serious test anxiety has to leave school to care for his/her ill mother. Therefore, this student is not able to take an exam that is necessary for passing the academic year. The only chance to still pass the academic year is to have his/her best friend talk to the exam committee as if he/she were the person with test anxiety. So, the friend has to convince the committee of the seriousness of the test anxiety by completing an anxiety-test and feign test anxiety. If the exam committee is not convinced of the test anxiety, the person with high test anxiety has to retake the whole academic year. Participants read the vignette and were then informed that if they were convincing in feigning test anxiety, they would take part in a lottery in which they could win an extra bonus of €20. After reading the vignette, participants filled in the SRSI again and had the MST once more. The participants did not receive any specific instruction how to respond to the task that followed. At the end of the second session, participants were asked to fill out a questionnaire. In this questionnaire, participants were asked to rate their understanding of the task; the plausibility of the vignette; and also their motivation, success, and their opinion about the task difficulty on a five-point Likert scale (anchors: 1 = Low; 5 = High). Our primary interest was in whether non-symptomatic participants exhibit the typical MST effect when they are instructed to feign. Because participants with raised anxiety levels might obscure MST results, we excluded students with BSI-18 scores that exceeded the cut-off.

### Statistical Analysis

Because our data were skewed, we used non-parametric tests, notably the *Wilcoxon Signed Rank* test and *r* for effect size using the formula proposed by Rosenthal (1994); *z/√N*(*number of observation*s) and *Mann–Whitney U-test*. According to Cohen (1988) criteria, the interpretation of *r* value is as follows: 0.1 = small effect, 0.3 = medium effect, and 0.5 = large effect. For clarity’s sake, we report means and standard deviations.

## Results

### Exit Questionnaire

Participants’ understanding of the task was high (*M* = 4.32, *SD* = 0.78), and they found the task moderately plausible (*M* = 3.77, *SD* = 0.75). Participants did not experience great difficulty in carrying out the task (*M* = 2.18, *SD* = 0.91). Furthermore, students were moderately motivated (*M* = 4.00, *SD* = 0.87), and they judged the success of their performance as rather modest (*M* = 2.77, *SD* = 0.61).

### Endorsement of SRSI Pseudosymptoms

From T1 to T2, there was a significant increase in endorsement of both genuine symptoms and pseudosymptoms, *Z* = 4.10, *p* < 0.001, *r* = 0.62, and *Z* = 4.11, *p* < 0.001, *r* = 0.62, respectively.

Importantly, at T2, the large majority of participants (*n* = 13; 77%) scored above the cut-off of nine pseudosymptoms. Looking at separate subscales of pseudosymptoms, it was apparent that the rise in pseudosymptoms was particularly evident for the Anxiety/Depression/PTSD subscale (*Z* = 4.12, *p* < 0.001, *r* = 0.62; see Supplementary Table 2). As to SRSI subscales that involve genuine symptoms, raised symptom scores during T2 were also particularly evident for the PTSD/Anxiety (*Z* = 4.13, *p* < 0.001, *r* = 0.62) and Depression (*Z* = 4.12, *p* < 0.001, *r* = 0.62). All in all, these significant increases in symptom endorsement indicate that our instructions to feign test anxiety at T2 were effective.

**Table 1** shows mean BSI-18 scores (only T1), mean SRSI (T1 and T2) scores, and Stroop latency data for the three categories of words (T1 and T2).

**Table 1:** Means and standard deviations of the BSI-18 scores (only T1), SRSI genuine symptoms and pseudosymptoms scores (T1 and T2), and MST latency data on neutral, anxiety, and test anxiety words in both conditions (*N* = 22).

Measures		T1	T2	Wilcoxon signed rank test	*r*
		*M (SD*)	*M (SD*)	*Z*	
BSI-18		3.77 (2.37)	/	/	/
SRSI	Genuine symptoms	6.82 (4.72)	31.27 (7.37)	4.10**	0.62
	Pseudosymptoms	1.23 (1.85)	15.64 (8.94)	4.11**	0.62
Stroop task (ms)	Neutral	512.95 (65.68)	745.63 (285.78)	3.65**	0.55
	Anxiety	517.10 (80.54)	795.95 (267.54)	3.94**	0.60
	Test anxiety	509.81 (75.68)	790.39 (286.01)	3.81**	0.57

*^∗∗^p < 0.001; T1, honest way of responding; T2, feigning test anxiety. BSI-18, Brief Symptom Inventory; and SRSI, the Self-Report Symptom Inventory.*

### The MST Effect

Response latencies during T2 were significantly longer than those during T1 (**Table 2**). This was true for neutral words: *Z* = 3.65, *p* < 0.001, *r* = 0.55, anxiety-related words: *Z* = 3.94, *p* < 0.001, *r* = 0.60, and test anxiety-related words: *Z* = 3.81, *p* < 0.001, *r* = 0.57.

**Table 2:** *Wilcoxon Signed Rank* tests (*Z*) and significance levels for differences in reaction times between different pairs at T1 and T2.

Pair of words	T1		T2		
	*Z*	*p*	*Z*	*p*	*r*
Neutral–anxiety	-0.50	0.61	2.84	0.005	0.43
Anxiety–test anxiety	0.27	0.78	1.28	0.200	0.19
Neutral–Test anxiety	1.35	0.18	2.06	0.039	0.31

*T1, honest way of responding; T2, feigning test anxiety.*

Comparing latencies between word categories revealed no significant differences at T1 (all *Z*’s < 1.35, all *p*’s > 0.18). However, at T2, differences emerged. The reaction time was significantly longer when anxiety-related words were presented compared with neutral words, *Z* = 2.84, *p* = 0.005, *r* = 0.43. The same pattern emerged when comparing latency for test-anxiety words with that for neutral words, *Z* = 2.06, *p* = 0.039, *r* = 0.31. The difference between anxiety and test-anxiety words remained insignificant (*p* = 0.20) (**Table 2**).

The magnitude of the MST effect at T2 (*M* = 44.75, *SD* = 92.65) was significantly larger than at the T1 (*M* = -3.15, *SD* = 31.72), *Z* = 2.06, *p* = 0.039, *r* = 0.31^3^.

Furthermore, we compared the MST effect at T2 between participants who scored above (*n* = 17) and participants who scored under the cut-point on the SRSI (*n* = 5). The MST effect in SRSI high scorers (*M* = 42.92, *SD* = 100.74) was not significantly raised from not detected feigners (*M* = 51.01, *SD* = 66.45), Mann–Whitney *U*-test = 38.00, *z* = -0.35, *p* = 0.724), but this might be due to the small sample size.

## Discussion

The MST has been proposed as a method for detecting pathology-related attentional bias (e.g., Becker et al., 2001). Some authors (Buckley et al., 2003; Constans et al., 2014; but see Thomas and Fremouw, 2009) have argued that the MST involves a reaction time pattern that is difficult to simulate and that therefore flags genuine psychopathology (e.g., PTSD). For example, Constans et al. (2014, p. 83) reasoned that it is “unlikely that someone who feigns PTSD symptoms could (1) correctly deduce that PTSD is associated with slightly slower responding on combat words, and (2) adjust color-naming response time to create the desired MST effect.”

The current study examined whether an MST effect can be obtained when non-symptomatic students are instructed to feign test anxiety. Our findings can be summarized as follows. First, looking into the SRSI scores, our feigning instructions were successful, meaning that they triggered the expected symptom over-endorsement pattern in feigning condition. That is, at T2 (feigning session), participants endorsed significantly more genuine and pseudosymptoms on the SRSI than at T1 (honest control session). The dominant strategy among participants instructed to feign test anxiety was to raise all symptom scores. However, symptom over-endorsement was particularly pronounced for anxiety-related symptoms. Furthermore, using the cut-off of 9, the SRSI pseudosymptoms subscale was able to detect 77% of participants in the feigning condition (T2).

Second, and most importantly, when participants were instructed to feign test anxiety (T2) without being informed about the MST effect, they produced longer reaction times for all three types of words. This overall slowing down likely reflects the increased cognitive load that is caused by feigning (see Vrij et al., 2008). However, a similar response pattern might be produced by patients, who are processing emotionally provocative material. Even though our study design limits us in discussing how would a genuine test anxiety group respond to the MST, we do know from the literature (e.g., Lawson, 2006) that the expected response pattern would be the MST effect. Some researchers, such as Buckley et al. (2002), even consider an overall delayed response latency to be a sign of genuine complaints. We disagree with this position. That is to say, genuine psychopathology might lead to delayed reaction times, but not each instance of a delayed reaction indicates psychopathology. For example, in one recent study (Boskovic et al., unpublished), we applied the MST to three groups: participants who had experienced high impact life events, participants with low impact life experiences, and actors, with low impact experiences, but coached to feign PTSD-related symptoms. We found the most delayed RTs in the actors (feigning) group, which opposes the position of Buckley et al. (2002). However, claiming that longer RTs can help detecting feigning, would be equally an overstatement of the case. The issue here is of course that people who feign symptoms on the MST are likely to produce longer response latencies (e.g., Boskovic et al., unpublished), yet people who exhibit longer response latencies are not necessarily feigning. For example, Becker et al. (2001) compared MST performance of people with generalized anxiety disorder (GAD), people with SP, and non-anxious participants. People with GAD had the overall longest reaction time while responding to both GAD-related and SP-related words.

Additionally, besides overall longer response latencies, participants specifically exhibited longer reaction times when responding to anxiety-related and test anxiety-related words compared with neutral words when feigning (i.e., the MST effect). This differential response pattern was not evident during the honest control session (T1). Thus, instructed students were able to simulate the MST effect in the feigning condition, indicating that this effect alone does not rule out feigning as previous studies claimed (e.g., Buckley et al., 2003; Constans et al., 2014). Our findings suggest that feigning and the cognitive load it induces (e.g., Cannon, 2003) might result in a response latency pattern (i.e., the MST effect) that closely resembles a response pattern that is widely considered to be a sign of genuine symptomatology. Cannon (2003) speculated that the MST effect reflects current concerns rather than authenticity of the complaints. This interpretation makes sense, given that people who are motivated to simulate a certain type of psychological problem will be preoccupied with key words referring to the features constituting that problem. The additional cognitive load caused by instructions to feign certain problems may in itself be sufficient for longer response latencies.

Even though both Buckley et al. (2003) and Constans et al. (2014) observed pronounced MST effects for trauma-related words in PTSD patients, there are several issues that preclude a straightforward interpretation of their findings. For example, Buckley et al.’s study relied on a small sample of actors, PTSD patients, and non-anxious controls (each *n* = 6; *N* = 18). The findings by Constans et al. (2014) actually fit nicely with our study, although the authors come up with a completely different interpretation of their results. The MST effect in their study was most pronounced in PTSD patients who were over-reporting symptoms on the M-FAST (Miller, 2001). Thus, it is a distinct possibility that the delayed reaction times on the MST in this group did reflect preoccupation with symptoms that were intentionally exaggerated rather than an attentional bias related to genuine PTSD.

A few limitations of the current study warrant comment. First, participants were tested during two different sessions, separated by a 1-week interval. This could possibly have resulted in a learning effect, because the tasks in the two sessions shared the same content. Participants had to react differently to the same tasks during the two sessions and we assumed that a learning effect was limited in this way. Still the within-subject design we employed might have influenced our results. However, if anything, one would expect that, during the retest, practice would have speeded up Stroop performance, thereby lowering the chances of finding a specific MST effect. If the learning effect did occur, then one can assume that the actual differences between the two conditions might be larger than what we observed. Second, we instructed students to feign test anxiety, a condition that will be relatively easy for them to relate to. Participants’ gratings of the difficulty to feign test anxiety indicated that students did not experience significant troubles in fabricating this particular issue. Whether students who are instructed to feign panic disorder, PTSD, or another less prevalent anxiety disorder also manifest an MST effect remains to be seen. Third, our findings are based on a non-symptomatic sample, therefore, we are restricted for making any conclusion regarding the responses of a genuine test anxiety group. We wanted to investigate whether providing a healthy, non-symptomatic sample with feigning instructions would produce a response pattern that is assumed to represent genuine test anxiety complaints (i.e., the MST effect). However, comparing feigners with a symptomatic group would be a logical and a necessary next step in investigating the meaning of the MST effect. It might well be the case that both groups exhibit MST effects, but that those in genuine patients are much higher than those in feigners. Finally, the employed within-subject design limited the in-depth analysis of the diagnostic utility of the MST (e.g., the ROC), which should be further explored. Future studies might also want to test patients instructed to feign certain conditions, which is not without ethical problems. Including people with genuine symptoms would imply that one would instruct half of them to deny problems and/or exaggerate problems, which is problematic. However, that would enable researchers to investigate more precisely the utility of both the MST and the SRSI in differentiating individuals who experience genuine symptoms from those who feign symptoms.

To sum up, we found that the MST effect could be relatively easy to elicit with feigning instructions (see also Cannon, 2003). Thus, the MST effect is not only sensitive to genuine psychopathology (Kimble et al., 2009), but also to the additional cognitive load (e.g., preoccupation of healthy people with certain themes and topics) caused by feigning, which introduces noise in the strength of this effect across clinical groups (e.g., people with PTSD). This may explain why Kimble et al. (2009) found that the MST effect is sometimes difficult to replicate. It might well be the case that our current understanding of the MST effect is hindered by a publishing bias (e.g., file drawer problem) (Kimble et al., 2009). Future work involving comparison of genuine patients and participants who feign is necessary in order to closely examine the quality and the utility of the MST effect in the symptom validity assessment. However, given the fragility of any MST-related findings, one needs to be careful in making diagnostic decisions only using isolated thresholds, such as reaction time.

## Ethics Statement

This study was carried out in accordance with the recommendations of the Manual application form by the Ethics Review Committee of the Faculty of Psychology and Neuroscience, Maastricht University with written informed consent from all subjects. All subjects gave written informed consent in accordance with the Declaration of Helsinki. The protocol was approved by the standing Ethical Committee of Faculty of Psychology and Neuroscience (ECP-159 03 12 2015).

## Author Contributions

IB is the main author and coordinator of this project. With the help and supervision of HM, LH, TM, and MJ, the first author created the idea and methodology of this study and wrote this manuscript. The author AB was engaged as assistant of this project and together with the first author helped collecting the data. All listed authors gave significant contribution to this paper.

## Conflict of Interest Statement

The authors declare that the research was conducted in the absence of any commercial or financial relationships that could be construed as a potential conflict of interest.

## Footnotes

1 Comparison of the Actors and Non-anxious group. Corresponding *r* value being 0.49.

2 A Dutch (original) version of the instructions can be obtained from the first author.

3 Due to the nature of the tests (ranks), the relative distance remains unchanged, hence, the same outcomes as for the MST effect at T2.
