# Supplementary material for: The Modified Stroop Task Is Susceptible to Feigning: Stroop Performance and Symptom Over-endorsement in Feigned Test Anxiety
Source: Front Psychol. 2018 Jul 11;9:1195. doi: 10.3389/fpsyg.2018.01195 (PMC6050504; doi:10.3389/fpsyg.2018.01195)
Supplement: Supplementary file 1 [file Table_1.DOCX]

**Supplemental file**

Supplemental Table 1. List of words used in the Stroop task. English translation with the original (Dutch) version in parenthesis.

| Type of words  English (and Dutch translation) | | |
| --- | --- | --- |
| Neutral | Anxiety – related | Test anxiety – related |
| Chair (Stoel)  Umbrella (Paraplu)  Solid (Massief)  Sitting (Zitten)  Walk (Wandeling)  Hammer (Hamer)  Pencil (Potlood)  Potato (Aardappel)  Locker (Kluis)  Shoe (Schoen)  Sock (Sok)  Water (Water) | Illness (Ziekte)  Injury (Verwonding)  Debts (Schulden)  Nervous (Nerveus)  Death (Dood)  Heartbeat (Hartslag)  Unemployed (Werkeloos)  Failure (Mislukking)  Abandoned (Achtergelaten)  Trembling (Huiveren)  Crazy (Gek)  Stroke (Beroerte) | Time (Tijd)  Test (Toets)  Panic (Paniek)  Worry (Bezorgd)  Exam (Examen)  Performance (Prestatie)  Frustration (Frustratie)  Doubt (Twijfel)  Confusion (Verwarring)  Challenge (Uitdaging)  Incomplete (Incompleet)  Incorrect (Incorrect) |

**Vignette (*English translation*)**

XX is 21 year old. S/he is a student at Maastricht University. XX lives in Maastricht in a student house. XX’s mother is currently living in another city. XX’s father died in an accident when XX was 10 years old. XX’s mother suffers from medical problems since then, so XX had to take care of her. XX started studies last year and came here after a caretaker had been arranged for mother. Currently XX is failing the year. XX suffers from high test anxiety, and this presents a huge obstacle for her/him. Therefore, XX was not able to do the tests in the previous period. XX has always had some problems with situations in which s/he was evaluated or judged, especially in test situations. However, this got worse over time, so now it is difficult for XX to take a test, because s/he starts panicking, sweating, and s/he fills like s/he is going to faint the minute the test starts. So, in this way XX failed a series of exams and (s)he has even running out all the resit possibilities. The only solution for XX to get an additional chance in retaking the exams is to go to the exam committee and tell them about the test anxiety problem. In order to succeed in getting a new chance for the tests, XX has to be very convincing in presenting the reasons for the privilege of extra resits.

Now, you are going to fill out the questionnaires about anxiety and related problems as if you were XX. Imagine that we are the exam committee and your questionnaires will be considered to be those of XX’s. If you succeed in convincing us that your questionnaire scores point in the direction of severe test anxiety, you will participate in a lottery with a 1: 10 chance of winning an extra bonus of 20 euro’s.

Now, we kindly ask you to read carefully the vignette again, and then start answering the questions as if you were XX.

Thank you and good luck!

| Supplemental Table 2. Means and standard deviations across conditions on genuine symptoms and pseudosymptom subscales on SRSI. | | | | | |
| --- | --- | --- | --- | --- | --- |
| SRSI |  | T1  *M*(*SD*) | T2  *M*(*SD*) | *Wilcoxon signed rank test*  *Z* | *r* |
| Credible symptoms | Cognitive | 1.27(1.67) | 6.18(2.36) | 3.93** | 0.59 |
|  | Depressive | .73(.88) | 5.54(1.94) | 4.12** | 0.62 |
|  | Pain | .82(1.10) | 3.32(2.64) | 3.60** | 0.54 |
|  | Non-specific somatic | 2.09(2.16) | 8.14(2.00) | 4.02** | 0.60 |
|  | PTSD / anxiety | 1.91(1.71) | 8.09(1.41) | 4.13** | 0.62 |
| Bogus symptoms | Cognitive/Memory | .36(.95) | 4.77(2.46) | 3.84** | 0.58 |
|  | Neurological: Motor | .18(.50) | 1.59(1.30) | 3.45** | 0.52 |
|  | Neurological: Sensory | .32(.72) | 2.86(2.33) | 3.69* | 0.55 |
|  | Pain | .14(.35) | 1.54(2.40) | 2.55** | 0.38 |
|  | Anxiety/Depression/PTSD | .23(.53) | 4.86(2.37) | 4.12** | 0.62 |
| Consistency check | | 3.59(1.47) | .82(1.01) | 3.97** | 0.60 |
| *Notes*: * *p* < .01; ** *p* < .001; T1 = honest way of responding; T2 = feigning test anxiety. | | | | | |
